# Insights on the Evolutionary Genomics of the *Blautia* Genus: Potential New Species and Genetic Content Among Lineages

**DOI:** 10.3389/fmicb.2021.660920

**Published:** 2021-04-26

**Authors:** José Luis Maturana, Juan P. Cárdenas

**Affiliations:** ^1^Centro de Genómica y Bioinformática, Facultad de Ciencias, Universidad Mayor, Santiago, Chile; ^2^Escuela de Biotecnología, Facultad de Ciencias, Universidad Mayor, Santiago, Chile

**Keywords:** *Blautia*, pangenome, phylogenomics, genomic species, diversity, gene gain/loss

## Abstract

*Blautia*, a genus established in 2008, is a relevantly abundant taxonomic group present in the microbiome of human and other mammalian gastrointestinal (GI) tracts. Several described (or proposed) *Blautia* species are available at this date. However, despite the increasing level of knowledge about *Blautia*, its diversity is still poorly understood. The increasing availability of *Blautia* genomic sequences in the public databases opens the possibility to study this genus from a genomic perspective. Here we report the pangenome analysis and the phylogenomic study of 225 *Blautia* genomes available in RefSeq. We found 33 different potential species at the genomic level, 17 of them previously undescribed; we also confirmed by genomic standards the status of 4 previously proposed new *Blautia* species. Comparative genomic analyses suggest that the *Blautia* pangenome is open, with a relatively small core genome (∼ 700–800 gene families). Utilizing a set of representative genomes, we performed a gene family gain/loss model for the genus, showing that despite terminal nodes suffered more massive gene gain events than internal nodes (i.e., predicted ancestors), some ancestors were predicted to have gained an important number of gene families, some of them associated with the possible acquisition of metabolic abilities. Gene loss events remained lower than gain events in most cases. General aspects regarding pangenome composition and gene gain/loss events are discussed, as well as the proposition of changes in the taxonomic assignment of *B. coccoides*^TY^ and the proposition of a new species, “*B. pseudococcoides.*”

## Introduction

*Blautia*, a taxonomic genus placed in the Lachnospiraceae family of the Firmicutes phylum, was initially described in 2008, from the reclassification of former *Ruminococcus* species isolated from stool samples (Liu et al., 2008). Members of this genus are obligately anaerobic, non-sporulating, coccobacillus-shaped Gram-positive microorganisms with the ability to ferment different carbohydrates. *Blautia* species are relatively abundant commensals in the healthy human GI tract, representing one of the top 10 most detected genera, representing between 2 and 8% of the human gut microbiome (Arumugam et al., 2011; Eren et al., 2015). *Blautia* species have a key role in indigestible carbohydrate degradation (Sheridan et al., 2016) and some of them, such *B. coccoides*, were shown to produce short-chain fatty acids (SCFAs), relevant metabolic mediators between the microbiota and the host (Liu et al., 2021). Additionally, some members had been proved to have more specific roles, as for example, *B. hydrogenotrophica*, a hydrogen consumer capable of establishing cross-feeding relationships with other members of the gut microbiota (Plichta et al., 2016). Microbes from this genus were associated with changes in diet, showing increased abundance under whole grain consumption (Martínez et al., 2013), or under a low-fat diet (Wan et al., 2019). *Blautia* genus is also found to be associated with a variety of physiological conditions. For example, *Blautia* members are associated with the production of health-promoting compounds such as SCFAs or antimicrobial peptides (Liu et al., 2021); additionally, *Blautia* species was found to be decreased in the microbiota of obese children, suggesting a potential role in the normal gut (Benítez-Páez et al., 2020). Opposite to this, there is also evidence for positive correlations between *Blautia* and diseases such as type I diabetes in children (Kostic et al., 2015), non-alcoholic fatty liver disease and non-alcoholic steatohepatitis in adult men (Shen et al., 2017), and chronic kidney disease (Barrios et al., 2015), among other conditions (Vacca et al., 2020). Since *Blautia* in general may be associated with positive and negative features in the human gut, the direct relationship between *Blautia*, health and disease are yet to be fully elucidated. This diversity of (positive or negative) associations may also reflect a previously unknown diversity among members of the genus, remaining to be discovered.

At this date, there are 17 officially accepted *Blautia* species, in addition to other 9 proposed so far, isolated from the GI tract from human and other mammals, including ruminants (Supplementary Table 1): *B. coccoides*, *B. hansenii*, *B. hydrogenotrophica*, *B. luti*, *B. producta*, *B. schinkii*, *B. wexlerae*, *B. glucerasei*, *B. stercoris*, *B. faecis*, *B. obeum*, *B. caecimuris*, *B. massiliensis*, *B. hominis*, *B. argi*, *B. brookingsii*, and *B. faecicola* (Liu et al., 2008; Furuya et al., 2010; Park et al., 2012, 2013; Lawson and Finegold, 2015; Lagkouvardos et al., 2016; Durand et al., 2017; Shin et al., 2018; Paek et al., 2019; Ghimire et al., 2020; Kim et al., 2020). In addition, there are nine proposed species “*Blautia marasmi*,” “*B. phocaeensis*,” “*B. provencensis*,” “*B. intestinalis*,” “*B. segnis*,” “*B. tardus*,” “*B. celeris*,” “*B. lentus*” and “*B. difficilis*” (Pham et al., 2017a,b; Traore et al., 2017; Liu et al., 2020). Currently, there are 225 non-redundant genomes available in RefSeq associated with *Blautia*, mostly obtained from isolates found in human samples (Supplementary Table 2); this dataset offers the opportunity to search for new previously undescribed species in this genus. The emergence of molecular systematics, based on the study of genome sequences as a source of taxonomic information, has offered the opportunity for the use of those undescribed genomes. For example, a recent analysis of 237 human-derived new Lachnospiraceae isolates (Sorbara et al., 2020) found previously unsequenced *Blautia* variants; additionally, the reconstruction of several genomes from the mouse GI tract (Lagkouvardos et al., 2016) detected the genome of a previously undescribed *Blautia* variant, describing *B. caecamuris* as a new species.

Despite the abundance and increased relevance of *Blautia* in the human microbiome, there is little information about its diversity and evolution. A very recent report of a *Blautia* pangenome was published (Liu et al., 2021), but no research on the diversity at the genomic level was made. The analysis of the currently available genomes, however, may reveal new insights about the evolution and development of potential *Blautia* species.

In this work, we present a pangenome analysis with a phylogenomic focus of a set of non-redundant *Blautia* genomes (available in the RefSeq database), in order to offer a first picture of the genomic diversity across the members of the *Blautia* genus, as well as to study the phylogenomic relationship among the lineages of *Blautia*. This study will also present a descriptive model depicting the main events involved in the development of different lineages across the genus.

## Materials and Methods

### Dataset Selection

A set of non-redundant *Blautia* genomes (available on September, 2020) was downloaded from the FTP sites from RefSeq genome repository from NCBI^1^. In order to ensure high-quality drafts, all the incomplete genomes were analyzed by CheckM (“lineage_wf” command) (Parks et al., 2015). Only genomes with completion of 95% or higher and contamination or heterogeneity lower than 5% were considered as “high-quality drafts” (as recommended in Bowers et al., 2017) and they were considered for further analyses. This analysis gave a final number of 225 selected *Blautia* genomes, specified in the Supplementary Table 2. From now, this reference genome set will be called the “*Blautia dataset.*” Sequences were analyzed by RPS-BLAST against the COG database (Tatusov et al., 2000), and by HMMer against PFAM (El-Gebali et al., 2019). The genomic data of *Robinsoniella peoriensis* DSM 106044 (GCF_005519995.1) was used as the outgroup in further analyses, since this taxonomic group seems to be the closest relative to *Blautia*, as been observed in both 16S rRNA and phylogenomic-based studies (Cotta et al., 2009; Hug et al., 2016).

### Pangenome Analysis

The pangenome reconstruction of the blautia dataset was performed with Roary (Page et al., 2015), panX (Ding et al., 2018) and PEPPAN (Zhou et al., 2020). For all the programs, input files were generated by prokka (default settings, *–kingdom Bacteria*) (Seemann, 2014). For Roary and PEPPAN, GFF files were used while for PanX, GenBank archives. Roary was run with ‘-e -n -p 24 -v -r -i 80 –group_limit 100000’ options. PEPPAN and PanX were run with default options. The output from PEPPAN was parsed using PEPPAN_parser with ‘-t -c -a 95′ settings. Using python scripts, the output of the previous step, namely allele.fna, PEPPAN.gff and PEPPAN.gene_content.Rtab, was used to generate a multifasta file containing the pangenome. The rarefaction curves for this pangenome were taken from the file PEPPAN.gene_content.curve and plotted using pandas (Reback et al., 2020) and matplotlib (Hunter, 2007). For PanX, the file geneCluster.json was parsed with pandas to generate a “presence and absence gene” matrix, to then obtain basic statistics about the pagenome. To estimate the pangenome openness/closedness, this matrix was fed into the R library micropan v2.1 (Snipen and Liland, 2015) and an alpha value was estimated.

### Functional Annotation of the Pangenome

The functional annotation of the pangenome defined by PEPPAN was carried out by eggNOG mapper v2.0.4-rf1 (Huerta-Cepas et al., 2017), using the eggNOG database version 5.0.1 (Huerta-Cepas et al., 2019). The annotations were transferred from the most specific taxonomic level out of *Blautia*, Clostridia, Firmicutes, or Bacteria (–tax_scope). Annotations from KEGG and COG databases were obtained for each gene. The percentages of each functional category were computed and plotted using pandas (Reback et al., 2020), seaborn and matplotlib (Reback et al., 2020). To assess the completeness of KEGG modules present in the pangenomes, MicrobeAnnotator was used in mode light (*–light*) with default options (Ruiz-Perez et al., 2021).

### TETRA, ANI, AAI Calculations and Definition of Genomic Species Groups

Pairwise comparisons for different genomes of the *Blautia* dataset were made by the calculation of pairwise ANI (average nucleotide identity), TETRA (tetra-nucleotide signature), and AAI (average amino acid identity) values (Chun et al., 2018). The ANI analysis was performed by FastANI (Jain et al., 2018); TETRA values were also calculated by using Jspecies version 1.2.1. (Richter and Rosselló-Móra, 2009). AAI values were obtained using the CompareM software^2^. From this data, we established how many clusters of genomic species are represented in the *Blautia* dataset, following a triple-condition criterium (see more details in Results), where it is required simultaneously that ANI > 95%, TETRA > 0.99, and AAI > 95%, to consider two genomes as members of the same genomic species group.

### Phylogenomic Trees

Gene families (Orthogroups) for the *Blautia* dataset plus the outgroup were obtained using OrthoFinder (Emms and Kelly, 2019) following default parameters. The protein sequences of all 190 conserved single-copy gene families found by OrthoFinder in the *Blautia* dataset plus the outgroup were retrieved using an in-house Perl script. Each orthogroup was aligned with MAFFT (L-INS-i mode) (Katoh et al., 2005), and the resulting alignments were concatenated and a partition file was created, where each partition corresponds to a single-copy orthogroup. For each partition, the best evolution model was computed using ModelTest-NG (Darriba et al., 2020) according to the Akaike information criterion (AIC) and passed to IQ-TREE 2 to build a species tree. IQ-TREE was used with 1000 replicates of ultrafast bootstrap (Hoang et al., 2018), optimizing UFBOOT trees by NNI (*–bnni*) and assessing branch support by a SH-like approximate likelihood ratio test (*–alrt*) (Guindon et al., 2010). In parallel, a single nucleotide polymorphism (SNP)-based tree for the core genome of the *Blautia* dataset was computed by the *panX* (Ding et al., 2018), using default parameters. The phylogenomic aminoacid and the SNP-based trees were compared using the function *cophylo* from the *phytools* library in R (Revell, 2012). When a taxa (i.e., a genome) showed an anomalous behavior in the tree (e.g., very long branches), its genomic classification to the *Blautia* genus was evaluated using the tool TypeMat, from the Microbial Genomes Atlas (MiGA) Online server (Rodriguez-R et al., 2015). If results reflected a taxonomic assignment different from *Blautia*, the genome was removed from the dataset.

Additionally, in order to depict the phylogenetic relationships between the different genomic species, an alternative version of the phylogenomic tree was computed used only a set of 35 genomes, representing the 33 genomic species (and two subspecies) found in the previous section (see Results section). This tree of representative genomes was made from the same set of 149 conserved single-copy gene families used in the aforementioned tree, following the same strategy described above.

### 16S rRNA-Based Comparisons

Sequences from reference strains found in Genbank (Supplementary Table 1), as well as all annotated 16S rRNA genes from the RefSeq genomes (Supplementary Table 2), were used as a reference database for comparison against all the 16S rRNA genes from the annotations found in the *Blautia* dataset. The sequences were compared using BLASTn, considering a cutoff identity value of 99% and minimum query and target coverages of 80%, as an indicator of a putative assignment to a species (for rRNAs with sequence length > 1400 nt), according to a previous report (Edgar, 2018).

### Gene Gain/Loss/Duplication Analysis

The set of representative genomes used for the representative tree (in addition to the outgroup) was used as the dataset for the calculation of the gene gain/loss model for *Blautia* species. The Orthofinder profile of gene families for the aforementioned 35 genomes was used by the software Count (Csurös, 2010) for the calculation of gene gain/loss rates following the Csûrös - Miklós model, optimized with a Poisson distribution at the root; the rates were also optimized considering a variation across families to 1:1:1:1 gamma categories for the edge length, the loss rate, gain rate, and the duplication rate, respectively. The convergence criteria were set to a likelihood delta of 0.1 with a maximum of 100 rounds. The calculated rates were used to generate an analysis following Wagner parsimony using the same penalty score for gains and losses.

### Detection of Horizontal Gene Transfer (HGT)

Genes potentially acquired by HGTs were inferred using HGTector v2.0b2 (Zhu et al., 2014). *Blautia* coding sequences were analyzed against a reference database of a set of 30 thousand RefSeq genomes (retrieved in March, 2021), formatted using DIAMOND (Buchfink et al., 2015). Valid hits (search) were obtained using default parameters. Sequences hits from species of the *Blautia* genus (NCBI TaxID 572511) were considered as self. Hits outside the genus were considered to be HGT hits.

## Results

### Defining Genomic Species Groups on the Basis of TETRA, ANI, and AAI Data

As a first step toward establishing clusters of species at the genomic level in the *Blautia dataset*, we calculated pairwise ANI (average nucleotide identity), TETRA (tetranucleotide frequency), and AAI (average amino acid identity) values for the entire genome dataset (Supplementary Table 3). The intraspecies boundaries were established as proposed before: pairwise comparisons showing ANI > 95%, TETRA > 0.99 (Richter and Rosselló-Móra, 2009), or AAI > 95% (Chun et al., 2018), were used to consider two genomes as members of the same genomic species. Scatterplots for each pairwise comparison between TETRA, AAI and ANI are represented in Supplementary Figures 1A–C. When those values were compared between each other in their capability to discriminate between inter- and intra-species, it was observed that > 99% of pairwise comparisons matched (represented by the purple dots in Supplementary Figures 1A–C). Only a small fraction of comparisons (for example, considering the TETRA vs ANI, or TETRA vs AAI plots) had only one of the criteria following the interspecies boundaries (red or blue dots, Supplementary Figures 1A–C). Additionally, density curves obtained from ANI, TETRA, and AAI data (Supplementary Figures 1D–F) showed that, in addition with the expected density peaks in the intra-species range, small density peaks in ANI and AAI data distribution just below the intra-species zone were detected. The presence of pairwise comparisons following just one of the two parameters, as well as the existence of the small density peaks below the intra-species range, raise the question whether ANI, AAI and TETRA values must be considered simultaneously to define genomic species groups. Since ANI is one of the most commonly used metrics used to define intra- or inter-species relationships between prokaryotic genomes (Richter and Rosselló-Móra, 2009; Jain et al., 2018), we will consider these values as the main parameter to define genomic species groups. In counterpart, TETRA and AAI will be used as secondary parameters, in addition with the percentage of identity of the complete (i.e., length > 1400 bases) 16S ribosomal RNA (rRNA) gene (Kim et al., 2014; Edgar, 2018).

Considering the aforementioned criterion, we found a set of 33 genomic species clusters from the *Blautia* dataset (Table 1 and Supplementary Table 4); twelve of those genomic species groups include one sequenced type strain: *B. argi*, *B. brookingsii*, *B. faecicola*, *B. hansenii*, *B. hominis*, *B. hydrogenotrophica*, *B. luti*, *B. massiliensis*, *B. obeum*, *B. faecis*, *B. caecimuris* and *B. wexlerae*. Additionally, we could find that four previously proposed new members can be considered as new genomic species (Liu et al., 2020): “*Blautia segnis*,” “*Blautia difficilis*,” “*Blautia intestinalis*” and “*Blautia celeris.*” These sequenced reference strains (from both official or proposed species) were useful to give a name to each group they represented (Table 1). In one group, we found genomes from representative strains from both official and a proposed species in a same genomic species group: the proposed species “*Blautia marasmi*” [strain Marseille-P2377, GCF_900258535.1, (Pham et al., 2017a)] was found to be part of the same genomic species group as *B. hominis* [strain KB1, GCF_002270465.1, (Shin et al., 2018)]. Moreover, we also found that both *B. producta* and *B. coccoides* type strains are part of the same genomic species cluster, since their pairwise values reflected this feature. The pairwise ANI, TETRA and AAI between the genomes of *B. producta* DSM 2950 (GCF_014131715.1) and *B. coccoides* DSM 935 (GCF_004340925.1) were 98.0877%, 0.99868, and 97.61%, respectively (Supplementary File 3). The implications of this genomic species clustering on a possible reclassification for *B. producta* and *B. coccoides* will be discussed later.

**TABLE 1:** Genomic species groups found in the *Blautia dataset* from the use of ANI, TETRA and AAI data.

Species cluster	Proposed species assignation	Included sequenced type strain?	# of genomes	Comments
1	*Blautia argi*	Yes	1	It includes type strain KCTC 15426 genome (GCF_003287895.1)
2	*Blautia brookingsii*	Yes	10	It includes type strain SG-772 genome (GCF_003011855.2)
3	*Blautia faecicola*	Yes	1	It includes type strain KGMB01111 genome (GCF_004123145.1)
4	“*Blautia segnis*”	Yes ^#^	2	It includes proposed type strain BX17 genome (GCF_014287535.1); representative rRNA sequence (MT905180.1) had 97.78% identity with NR_109014.1 (*Blautia faecis* M25)
5	[unknown group A]	No	1	Representative rRNA sequence (locus B5F53_RS19410) had 92.88% identity with NR_026312.1 (*Blautia schinkii* strain B), so it could be a previously undescribed species
6	[unknown group C]	No	1	The only representative does not contain a suitable 16S rRNA gene
7	[unknown group D]	No	1	Representative rRNA sequence (locus_tag G5B11_RS18600) had 95.44% identity with *B. glucerasea* strain JCM 17039 16S rRNA (NR_113231.1), so it could be a previously undescribed species
8	[unknown group E]	No	1	Representative rRNA sequence (locus_tag G5A70_RS15400) had 97.45% identity with *B. glucerasea* strain JCM 17039 16S rRNA (NR_104687.1), so it could be a previously undescribed species
9	“*Blautia difficilis*”	Yes ^#^	1	It includes the proposed type strain M29 genome (GCF_014297245.1)
10	*Blautia hansenii*	Yes	1	It includes type strain DSM 20583 genome (GCF_002222595.2)
11	*Blautia hominis*	Yes	3	It includes the genomes of *B. hominis* type strain KB1 (GCF_002270465.1) and the proposed “*B. marasmi*” proposed type strain P2377 (GCF_900258535.1)
12	*Blautia hydrogenotrophica*	Yes	3	It includes type strain DSM 10507 genome (GCF_000157975.1)
13	*Blautia luti*	Yes	1	It includes type strain DSM 14534 genome (GCF_009707925.1)
14	“*Blautia intestinalis*”	Yes ^#^	5	It includes proposed type strain 2744 genome (GCF_014297355.1)
15	[unknown group B]	No	1	The genome of the unique member of this clade was labeled as “*B. caccae*” (unofficial name). The representative rRNA sequence (locus_tag BQ7352_RS08285) had < 98.5% identity with AB534168.1 (from *B. hansenii*).
16	*Blautia massiliensis*	Yes	25	It includes type strain GD9 genome (GCF_001487165.1)
17	[unknown group F]	No	2	Representative RNA genes had < 98.5% identity with other RNA genes from known species. This suggests that it could be a new species
18	*Blautia obeum*	Yes	36	It includes type strain ATCC 29174 genome (GCF_000153905.1)
19	*Blautia producta* (subspecies *coccoides* and *producta*)	Yes	5	It includes *B. coccoides* type strain DSM 935 (GCF_004340925.1) and *B. producta* type strain DSM 2950 (GCF_014131715.1) genomes
20	[unknown group J]	No	6	Representative RNA genes had <98.5% identity with other RNA genes from known species. This suggests that it could be a new species
21	[unknown group G]	No	4	Representative RNA genes had <98.5% identity with other RNA genes from known species. This suggests that it could be a new species
22	[unknown group H]	No	2	Representative RNA genes had <98.5% identity with other RNA genes from known species. This suggests that it could be a new species
23	[unknown group K]	No	2	Representative RNA genes were very close to other strains, but <98.5% identity. This suggests that it could be a new species
24	*Blautia faecis*	No	5	Representative rRNA sequences (e.g., locus G4470_RS18450) had >99% identity with *B. faecis* type strain 16S rRNA sequence (NR_109014)
25	[unknown group L]	No	3	Representative RNA genes had <98.5% identity with other RNA genes from known species. This suggests that it could be a new species
26	[unknown group I]	No	23	Representative RNA genes had <98.5% identity with other RNA genes from known species. This suggests that it could be a new species
27	*Blautia caecimuris*	No	2	Representative rRNA sequence (locus G4948_RS15000) had 99.35% identity with *B. caecimuris* type strain 16S rRNA sequence (KR364746.1)
28	“*Blautia celeris*”	Yes ^#^	5	It includes strain NSJ-34 genome (GCF_014287615.1); representative rRNA sequence (MT905182) had 98.26% identity with NR_163637.1 (*Blautia hominis* strain KB1), so it could be a previously undescribed species
29	*Blautia wexlerae*	Yes	66	It includes strain DSM 19850 genome (GCF_000484655.1)
30	[unknown group X]	No	2	Representative rRNA sequences from this group have > 99% identity with type strain *B. wexlerae* sequence (NR_044054), despite rRNA sequences are smaller than 1300 nt. It may constitute a “gray zone” subspecies.
31	[unknown group Y]	No	3	It includes *B. coccoides* YL58, with rRNA sequences having < 98% identity with sequences from several other species.
32	[unknown group W]	No	1	Representative RNA genes were very close to *B. obeum* type strain sequence, but < 98% identity. This suggests that it could be a new species
33	[unknown group Z]	No	1	Representative RNA genes had < 97% identity with other RNA genes from known species. This suggests that it could be a new species

*# Contains a genome from a representative strain for a non-confirmed species.*

Several groups did not include any sequenced type (or representative) strains. In some of these cases, the analysis of the 16S rRNA gene sequence encoded by those genomes was useful for the assignment of a species name to the members of the cluster. For example, in the case of the *B. caecimuris* cluster (cluster #27, Table 1), the name was assigned since the 16S rRNA gene from those genomes were > 99% identical to the *B. caecimuris* type strain 16S rRNA sequence (KR364746.1). Additionally, despite no type strain of *Blautia faecis* is sequenced, members of cluster #24, composed by 5 genomes (Table 1), contain 16S rRNA sequences with high identity (>99%) with the sequence from *B. faecis* strain M25^T^ (NR_109014), so this cluster can be assigned to the aforementioned species. Unfortunately, potential genomes representing “*B. phocaeensis*,” “*B. provencensis*,” *B. schinkii*, *B. glucerasei*, and *B. stercoris* were not found by this strategy.

It was also noticeable that, in some cases, the genomic species clustering gave strange cases in the separation between species, creating some “gray zones.” For example, the case of genomic species cluster #31 (Table 1), containing a strain called *B. coccoides* YL58 (GCF_002221555.2), is characterized to have representative 16S rRNA gene sequences with high identity (∼ 99%) to sequences from *B. wexlerae* DSM 19850 (NR_044054.1), despite the representative sequences from those genomes (*locus_tag* G4422_RS16765 for *B. wexlerae* MSK.20.14 and *locus_tag* G4417_RS16440 for *B. wexlerae* MSK.20.46) are only 1198 and 1273 bases long respectively, and the high identity may be an artifact caused by the incomplete rRNA sequence length. Additionally, despite that this genome had ANI values lower than 95%, but TETRA values higher than 0.99, in comparison with genomes from cluster #11 (Supplementary Table 4). In this case, the use of a more complete version of the 16S rRNA gene for this strain (KR364747, 1521 bases) was useful to show potential relatedness to other genomic species groups. The best match against the rRNA genes from other members of the *Blautia* dataset was a sequence from *Blautia* sp. NSJ-34 (cluster #28), with 98.75% identity, a value below the recommended 99% identity proposed for operative taxonomic units (Edgar, 2018). This suggests that this genome (and their genomic species group) was wrongly assigned to *B. coccoides* and it may be part of a previously undescribed species.

Considering the previous results, our strategy suggests the existence of 17 additional genomic species clusters in the *Blautia* dataset corresponding to new, potentially undescribed species, labeled as “unknown groups”. It was quick to notice that many of those genomes had incorrect name assignments (e.g., strain 1001175st1_E1 assigned to *B. hansenii*, being part of the *B. caecimuris* cluster), making evident the need of a careful curation of the taxonomic assignment for sequenced strains. Some of the unknown groups contain multiple genomes, such as Group I, with 23 genomes, or Group J, with 6. Some groups could have previously assigned names, even if no formal proposal was made: one of them, called “unknown group B” (represented by 1 genome, *Blautia* sp. Marseille-P3201) was labeled as “*Blautia caccae*” in their respective project (NCBI Bioproject PRJEB18018), despite no study is currently available about this potential species name assignment. Additionally, in those clusters, the information of 16S rRNA gene identity search (against the reference database) could not give conclusive results for a species-level taxonomic assignation (e.g., by giving identity lower than 99% in 16S rRNA gene comparisons), opening the possibility that all those groups may represent currently new, unnamed *Blautia* species.

As a final addendum, it was observed that the assembly of *Blautia* sp. M16 (GCF_014287855.1, See Supplementary Table 2), proposed in a preprint as another representative of a new species (“*Blautia lentus*”) (Liu et al., 2020), was not included in the final list. A posterior BLAST-based analysis with the predicted proteome of M16 strain showed that 86% of the best hits for each protein was found from a member of the Clostridiaceae family, despite the fact that one of the copies of the 16S rRNA gene have a closer relationship with members of *Blautia* (data not shown). Additionally, the use of MiGA showed that this genome cannot be classified as a *Blautia* on the basis of the genome content (data not shown). This suggests that this genome corresponds to a member of a Clostridiaceae rather than to a member of the *Blautia* genus (Lachnospiraceae family). Therefore, this genome was not considered as part of the *Blautia dataset*.

### Phylogenomic Analyses

**FIGURE 1:**
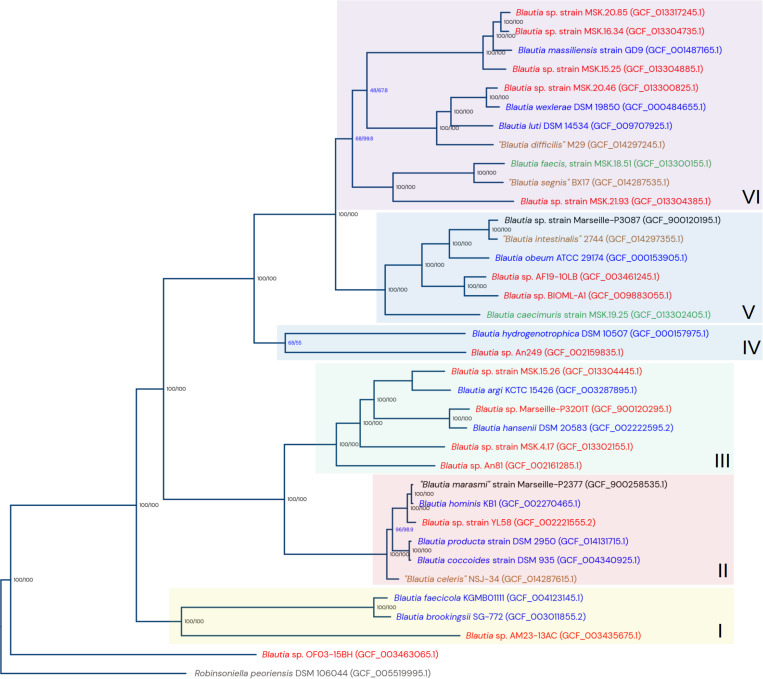
Phylogenomic tree from selected representative genomes from the clusters of genomic species from the *Blautia* dataset. The tree was computed using maximum likelihood, from the concatenated sequence alignment of 190 conserved single-copy orthogroups. The numeric data in format *n/n* represent values for bootstrap support (UF-bootstrap) and approximate likelihood-ratio test values (SH-aLRT). The tree is rooted using *Robinsoniella peorensis* data as the outgroup. The arbitrarily designed lineages I to VI are also shown. The color of each taxon name reflect the status of the current representative genome: red: undescribed potential new species; blue: type strain of a validated species; green: strain of a validated species, but not a type strain; brown: proposed type strain from a non-officially accepted species; black: current strains reassigned into another species. See text for more details.

After obtaining the clusters of genomic species, the next step in the analysis was the reconstruction of a phylogenetic tree to obtain a picture of the evolution of the *Blautia* genus. From a set of 190 single-copy orthogroups conserved among the genomes from the *Blautia* dataset and the outgroup (obtained using Orthofinder), a phylogenomic tree was constructed using IQTREE (Supplementary Figure 2). Additionally, using the conserved SNP set from the single-copy conserved genes found by panX, we constructed another phylogenomic tree (Supplementary Figure 3); both trees gave important visible correlations with each other (Supplementary Figure 4). In parallel, in order to simplify the visualization of the different species lineages detected in the *Blautia* dataset, another tree was computed with the same data from the main tree, but with only 35 taxa, corresponding to selected representatives from each genomic species cluster (Figure 1). This representative taxa tree had the same genomic species relationships as the main tree. The closest genome species group to the established root is the unknown Group C. The rest of the taxa were divided into six clades (numbered by roman numbers in Figure 1). The nodes representing the last common ancestor (LCA) for each lineage were defined considering the depth of each selected node, as well as the distance of those nodes with respect to the root of the genus (data not shown).

The lineage I contains the genomic species *B. faecicola*, *B. brookingsii*, and the “unknown group I.” The lineage II contains the species *B. hominis* (including “*B. marasmi*” as a subspecies), the new group *B. coccoides-producta*, the non-officially accepted “*Blautia celeris*,” and the “unknown group Y”. The lineage III contains the genomic species *B. argi*, *B. hansenii* and the unknown groups E, B, L, and H. The lineage IV contains *B. hydrogenotrophica* and the “unknown group A”. Lineage V contains *B. caecimuris*, *B. obeum*, “*B. intestinalis*,” and the unknown groups F and G. Finally, the lineage VI includes *B. massiliensis*, *B. luti*, *B. wexlerae*, *B. faecis*, “*B. segnis*” and the unknown groups D, J, K, X, and Z. Clades II and III share a directly common ancestor, as well as clade V with clade VI.

Almost all branches are supported by both optimal bootstrap and aLRT values (i.e., 100/100), with the exception of the *B. hydrogenotrophica* - “unknown group A” bifurcation (lineage IV, Figure 1), and two bifurcation events in the lineage VI. Interestingly, in the major tree (Supplementary Figure 2), the branches with suboptimal bootstrap and SH-aLRT values represent the same taxa and genomic species clusters observed in the representatives’ tree. Additionally, when the concatenated protein families and the SNP-based major trees were compared (Supplementary Figure 4), the main discrepancies were found at the intra-species level (where branch support values are now shown), and in the aforementioned suboptimal branches. Despite those described low support values, the direct bifurcation events between the different genomic species are generally well supported, with the exception of the bifurcation between the *B. hydrogenotrophica* cluster and the “unknown group A,” which have long branches and just moderate value: 68/55 in the representative tree (Figure 1), 48.9/63 in the main tree (Supplementary Figure 2). Both the bifurcation and its tendency in having long branches, are also observed in the main tree. This may reflect that this lineage is particularly divergent from their brother clades.

### Definition of a Pangenome for the *Blautia* Dataset

Given the myriad of software pipelines for pangenome reconstruction available (over 40) (Vernikos, 2020), we assessed three different pipelines to define the pangenome of the *Blautia* dataset: Roary (Page et al., 2015), panX (Ding et al., 2018) and PEPPAN (Zhou et al., 2020). Roary and panX, well-established packages based on their number of citations and publication dates, define clusters of homologous proteins in a broadly similar way. The main difference lies in the way they establish clusters of orthologs proteins. Roary uses conserved gene neighborhood information, splitting clusters of homologs sequences into paralogs and true orthologs. On the other hand, panX builds phylogenies of the homologous clusters and splits them into clusters of orthologous sequences by examining the structure of these trees. PEPPAN, the most recent software out of the three (2020), uses the information from gene cluster trees and synteny of these genes to split paralogs from orthologs groups.

All the software used here defines the pangenomes as the total set of clusters of orthologs, or orthogroups, present in all the genomes. In general terms, the number of orthologs per category found by PEPPAN and panX are similar, while Roary results depart from these (Table 2). Roary defined the pangenome as composed of almost 90 thousand orthogroups, approximately double compared to the other two programs tested. Furthermore, PEPPAN and panX found 821 and 722 core orthogroups respectively, i.e., the set of orthogroups that have at least one ortholog present in all the genomes (Table 2), while Roary only found 117 core orthogroups.

**TABLE 2:** Number of genes per pangenome category.

Program	Core genes	Soft core genes	Shell genes	Cloud genes	Total genes
Peppan	821	136	4452	38136	43545
PanX	722	173	3433	31109	35437
Roary	117	36	5875	82534	88562

*#: Number of strains. Core: 99% ≤ # ≤ 100%; Soft core: 95% ≤ # < 99%; Shell: 15% ≤ # < 95%; Cloud: 0% ≤ # < 15%.*

**FIGURE 2:**
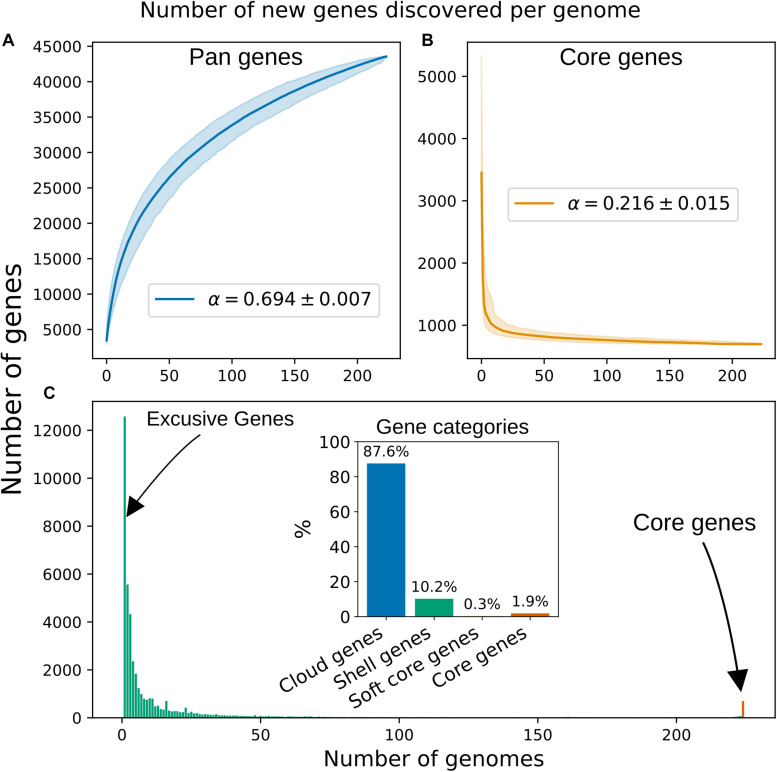
Rarefaction curves for “pan” **(A)** and “core” **(B)** genes. The curves were fitted to median values of 1,000 iterations following the Power Law previously described (Tettelin et al., 2008). The Shadows indicate 95% confidence intervals. **(C)** Distribution of the 43545 pan genes among the 224 genomes of the *Blautia* dataset. The bar representing the core genes is highlighted with red color. Inset: Percentages for each gene category in the pangenome, see Table 2 for definitions.

Using the pangenome defined by PEPPAN, we computed rarefaction curves and fitted the data according to a Power Law (Tettelin et al., 2008), from which an alpha value was computed. An alpha value <1 is considered as an indication of an “open” pangenome, which suggests that new orthogroups will be found if new genomes were added. We computed an alpha value of 0.694, suggesting that the pangenome of the *Blautia* dataset is “open” (Figure 2A). We carried out the same calculation using the pangenome defined by panX and found an alpha of 0.518, a roughly similar value, which was expected given that both programs reconstructed similar pangenomes in terms of their “orthogroups presence and absence” matrix. The pangenome rarefaction curve (Figure 2A) predicts that approximately 72 orthogroups are gained per new genome added, while the core genome curve (Figure 2B) implies that approximately 0.62 core orthogroups are lost per new genome added. In terms of the distribution of the genes among the 224 genomes analyzed (Figure 2C), we can see a high peak of genes shared by 10 or fewer genomes. When looking at the flat part of the histogram, which encompasses most of the “accessory” genome (here, represented roughly by genes shared by at least 50 genomes but no more than 200), we found that the average number of shared genes was 18.

### Functional Annotation of the Pangenome

The pangenome reconstructed by PEPPAN was functionally annotated with eggNOG-mapper (Huerta-Cepas et al., 2017). Additionally, KEGG annotations and assessment of pathways completeness were performed by MicrobeAnnotator (Ruiz-Perez et al., 2021). According to eggNOG-mapper, 32755 genes obtained any kind of functional annotation, i.e., COG, KEGG, GO or PFAM, of which 85.6% entries were assigned at least one COG category. For the core genome (Supplementary Figure 5) the *Metabolism* meta-category was the most represented, with 35%, under which transport and metabolism of coenzymes, amino acids and nucleotides together with *Energy production* categories were the most prominent, making up 74% of this meta-category. For example, in the carbohydrate metabolism, the glycolysis (Embden-Meyerhof pathway, M00001) was present with 100% completeness and the glycolysis core module (3C compounds) with 90% completeness (M00002). Furthermore, the Pentose Phosphate pathway (M00007) and glycogen biosynthesis were present with 100% completeness. When looking at ATPases, it is worth noting that the V-type ATPases are 80% completeness in the core genome, i.e., most of its 9 subunits are present in 98% of the genomes or more, meanwhile the F-type ATPase is not part of the core genome, indicating that some members of the *Blautia* data set do not have any subunit.

**FIGURE 3:**
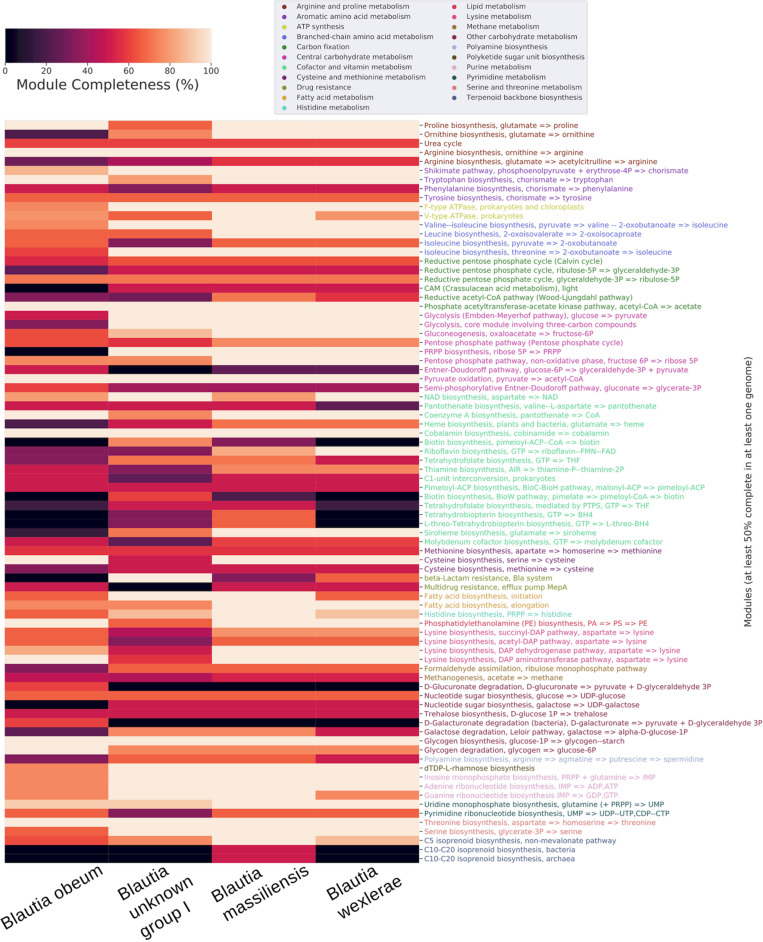
Heatmap of the KEGG modules completeness of the core pangenomes of 4 selected genomic species groups, clustered by category of metabolism. All clades have at least 23 genomes and are composed of genomes pertaining exclusively to one cluster defined by ANI. Only modules with 50% completeness or more were considered.

In order to present a more detailed view of the *Blautia* metabolism, we selected 4 clades from the complete tree and using PEPPAN, built a pangenome for each one. All clades have at least 23 genomes and are composed of genomes pertaining to only one cluster defined by ANI. Clades correspond to *Blautia* unknown group I, *Blautia obeum*, *Blautia massiliensis* and *Blautia wexlerae* respectively (highlighted clades from Supplementary Figure 1 and Figure 3).

When analyzing the core genomes of each selected clade, multiple pathways in the pentose phosphate cycle displayed differences in the core genome of *Blautia sp.* group I cluster compared to the other 3 clades. For example, the biosynthesis of Phosphoribosyl pyrophosphate (PRPP, M00005), essential in *de novo* pathways for synthesis of pyrimidines and purines, it is not present in the core genome of *Blautia sp.* group I clade but It was present with 100% completeness in the rest of the clades analyzed. On the other hand, the enzyme 2-dehydro-3-deoxyphosphogluconate aldolase, part of the Entner-Doudoroff pathway, d-glucuronate and d-galacturonate degradation pathways, which catalyzes the reaction from 2-Dehydro-3-deoxy-6-phospho-D-gluconate to d-glyceraldehyde 3-phosphate (releasing pyruvate), which in turns can be converted to glycerate-3P and then enter glycolysis, was present exclusively in the core genome of the *Blautia sp.* group I clade. The two glucoronate degradation pathways mentioned earlier were represented partially with the enzymes glucuronate isomerase (K01812), which can catalyze the conversion between glucoranate/galactunorate to fructosa or other pentoses which are able to continue the phosphate pentose pathway, and 2-dehydro-3-deoxygluconokinase (K00874), which phosphorylates deoxy-d-gluconate to continue to form pyruvate and glyceraldehyde as final products.

The Shikimate pathway, which produces chorismate, precursor of the aromatic amino acids, is 100% complete in all the clades except in the clade corresponding to *Blautia* sp. group I, where it is 86% complete. These species may be seen as an exception to what has been reported with respect to the existence of this pathway in the human gut microbiome, where most of the bacteria present in fecal samples do not possess a complete Shikimate pathway (Mesnage and Antoniou, 2020). Regarding the pathways involved in the biosynthesis of the aromatic amino acids, the pathway for tryptophan was complete in the 4 clades but tyrosine and phenylalanine pathways were 60% or less complete. Related to this, it has been shown that despite the fact that the Shikimate pathway may be complete in some metagenomes of the human microbiome, the metratrascriptomes of these pathways were largely inactive, suggesting that most of the microbiome bacteria are aromatic amino acid auxotrophs (Mesnage and Antoniou, 2020).

In respect of resistance to some metabolites, it was observed that the multidrug resistance efflux pump MepA set of proteins is 50% complete in the core genome of all the clades except in the one corresponding to *Blautia obeum*. On the other hand, *Blautia* sp. group I presented 100% completeness of the set of proteins forming the beta-Lactam resistance system, whereas in the rest of the clades it was absent.

### Gain/Loss Patterns Among *Blautia* Organisms

**FIGURE 4:**
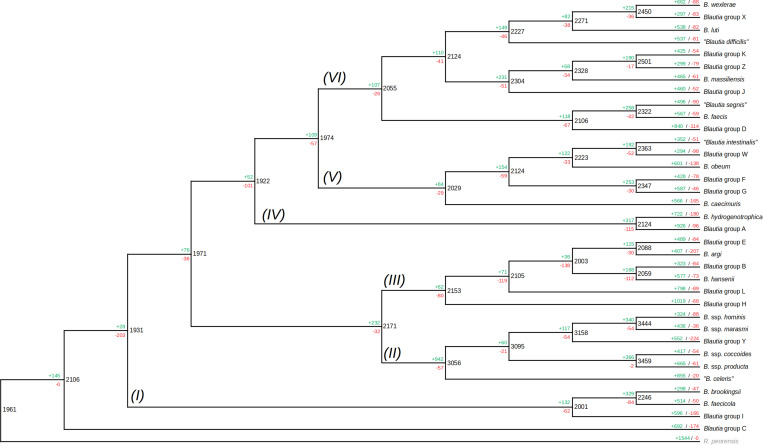
Gain/loss profile among different ancestors and lineages among the representative set of *Blautia* genomic species. The same tree (with the same members) shown in Figure 1, was presented as a cladogram, and the data from Count analysis of gene gain/loss models, following Wagner parsimony model for 12,691 gene families, was represented. The roman numbers near each branch represent each proposed lineage among the members. Black numbers represent the number of shared families for a given node (i.e., predicted ancestor), as well as the green and red numbers represent the number of gene families gained and lost for the ancestor. The green and red numbers on the upper side of each terminal branch represented the same terms as before, but for the members of the tree (the terminal nodes). See text for more details.

The pangenome of *Blautia* members was shown to be open. However, the evolution of the gene content among genomes from this genus remains to be elucidated. In order to predict the history of gene family gain and loss events among the different *Blautia* lineages, the gene family profiles (including gene families found in only one genome) were used to generate a gene gain/loss model in the *Blautia* set of representatives (Figure 4) using Wagner parsimony analysis with the Count tool (assigning equal weight to losses and gains). Considering the same 35 organisms utilized in the tree from Figure 1, a set of 2106 gene families predicted to be found in the LCA of *Blautia*. According to COG assignments, 13.95% of the predicted genes from the *Blautia* LCA, were not assigned to any category. From this same set of 2106 families, 32.92% of these genes were associated with Metabolism; 19.19 and 17.33%, of these genes were associated with Information storage and processing processes and Cellular processes and signaling, respectively, and COG families from “Poorly characterized” categories covered 16.61% of assignments.

In comparison with the outgroup (*R. peoriensis*), the *Blautia* LCA shares 1961 genes; whereas *Blautia* LCA acquired 145 genes in comparison with the predicted ancestor with the outgroup, *R. peoriensis* had 1544 genes that were not detected in the *Blautia* LCA. Among the 145 genes acquired by the *Blautia* LCA, 23.84% of them were assigned to Metabolism-associated functions and 23.18% of those genes were unassigned to any COG. The presence of gene family gain events in the predicted ancestors, as well as a high percentage of genes associated with Metabolism in those acquired families will be common trends in the next predicted gene gain events (see below).

COG classification for the acquired genes in each lineage (Figure 4 and Supplementary Figure 6) showed that, whereas massive gene gain events were observed in the terminal nodes (i.e., when genomic species were already defined), a noticeable amount of gene gain events were also observed in some of the internal nodes of the tree (i.e., the predicted ancestors). For example, it was observed that the LCA of the lineage II received 942 gene families, becoming the most massive gene gain event observed in any predicted ancestor of the reference set. Another important gene gain event observable in the internal nodes in the case of the LCA between *B. hydrogenotrophica* and the “unknown group A” representative (lineage IV), receiving 327 gene families. Gene loss events were not as massive as gene gain events, but there are some cases worth noticing. For example, it was observed that the predicted LCA between the deeply branched Group C and the rest of *Blautia* (Figure 4) suffered the loss of 203 families. From those missing genes, 29.33% of them did not have any COG, and 29.81% were involved in Metabolism, including sets of subunits of ABC transporters and some glycosyl hydrolases. Despite this particular case, however, gene loss seemed to be a secondary strategy used in the evolution of the *Blautia* species, in comparison with gene gain events.

From the 942 genes gained in the lineage II LCA, 34.05% were found to be associated with Metabolism, 13.37% with Cellular processes and signaling functions, and 11.51% with Information storage and processing. In the case of genes acquired in the LCA of the lineage IV, 28.4% were associated with Metabolism, 14.8% with Cellular processes and signaling functions, and 9.06% with Information storage and processing (Supplementary Figure 7). In both cases, the percentages of gene families unassigned to any COG were 25.37 and 33.84% respectively. Some remarkable functions found to be acquired by the lineage II are a set of enzymes for ammonia assimilation (glutamate dehydrogenase and glutamine synthetase), different subunits of ABC transporters, the aerobic-type carbon monoxide dehydrogenase complex, the rubrerythrin, several potential transcriptional regulators, the pyruvate ferredoxin oxidoreductase complex, a butyrate kinase (final enzyme in a butyrate biosynthesis pathway), etc.; those genes may be involved in the improvement of the metabolic program of the new lineage. Some examples of gene functions gained in the lineage IV LCA were several subunits of the archaeal/vacuolar-type H + -ATPase, several other subunits from ABC transport systems, and different proteins involved in oxidative stress response (peroxiredoxin, thioredoxin reductase, and another rubrerythrin). Expectedly, this differential gained gene content reflects the differential adaptation of the lineages during their respective evolutive processes.

In the case of the terminal nodes of the gene gain/loss model tree (i.e., reflecting the most recent gene/loss events in the observed genomes), the tendency to have more gene gain than gene loss events was also observable. Gene gain event numbers fluctuate between less than 30, to even more than 1000 genes (see *Blautia* group H terminal node, Figure 4). According to the profile of COG assignments from gained and missing genes (Supplementary Figure 7), near 62% of the total genes acquired in the terminal nodes were unassigned to any COG (as well as a 9% of “Poorly Characterized” COG functions), and just 13.43% of the acquired genes were assigned to COG associated with Metabolism. In counterpart, 30.59% of the missing genes in terminal nodes were related to Metabolism and only 24.82% were unassigned to COGs. In the set of acquired genes in the terminal nodes, several genes associated with DNA transfer functions (such as prophage components, CRISPR-associated proteins, or plasmid-related genes) were detected (data not shown), suggesting important part of the acquired genes in the terminal nodes are involved in DNA mobility or gene exchange processes. As expected, most of the gained genes had the label of “hypothetical protein.” These acquired genes clearly correspond to the cloud pangenome observed in previous sections, representing the most recently acquired gene set from each one of the representative genomes.

**FIGURE 5:**
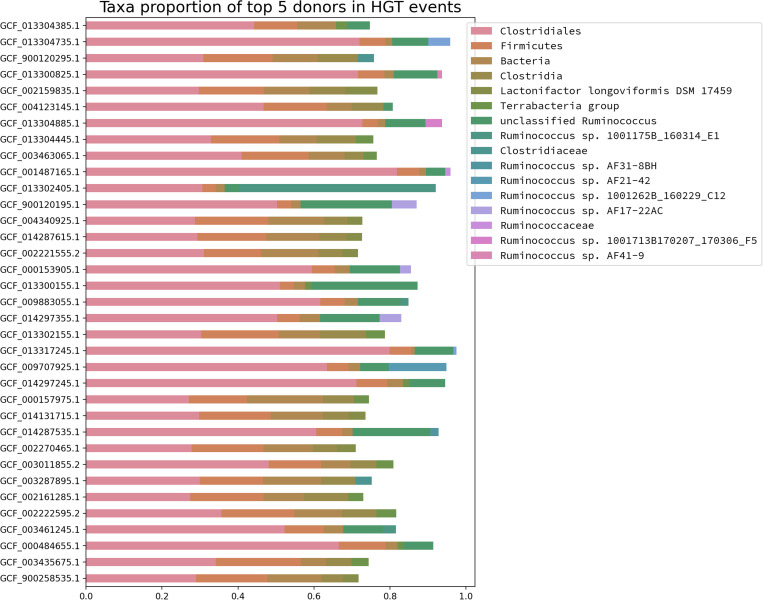
Taxonomic abundance profile of the top five HGT donors for the *Blautia* genomes from the representative dataset. Each genome was analyzed by HGTector and the frequency of taxonomic assignments for the top 5 putative donors were plotted for each case. The color code corresponds to the donors listed in the legend (upper right side).

The detection of massive gene gain events among different predicted *Blautia* LCA raised the question about where those acquired genes come from. In order to address this question, the predicted proteomes from the *Blautia* representative dataset were analyzed by HGTtector, a tool that makes HGT predictions based on the analysis of hierarchies of sequence alignment hits distributions, given a representative database (Zhu et al., 2014). If the top 5 donor taxonomic groups, represented for each genome from the representative *Blautia* dataset, were plotted (Figure 5), it is noticeable that for almost all cases, the most important predicted donor for those *Blautia* genomes is a member of the Clostridiales order (range: 81–27% of genes), followed by a member of the Firmicutes phylum (range: 22–3.6%). In some cases (up to ∼20%), ŁHGTector could only assign a potential donor to a generic member of the Bacteria superkingdom. It is also worth noticing that several *Blautia* genomes have predicted HGT donors Łfrom more specific taxonomic groups such as a variety of *Ruminococcus* sp. strains, most of them isolated from the human microbiome (Zou et al., 2019), or *Lactonifactor longoviformis*, a member of the Clostridiaceae family, also isolated from human stool samples (Clavel et al., 2007). When HGTector data was intersected with the Count analysis results, it was found that between 30 and 40% of the gained fraction among *Blautia* lineages were also found to have a putative HGT donor by HGTector. Among those transferred genes, a remarkable example of an acquired functional gene set corresponds to a group of 4 genes, gained by the LCA of the Lineage II (but absent in the LCAs from the others), encoding a functional module for the oxidative branch of the pentose phosphate pathway (KEGG module M00006), putatively transferred from a member of Clostridiaceae (data not shown). In the LCA from Lineage II also were found 6 genes encoding components of the “Pentose phosphate pathway, non-oxidative phase” module (M00007, 50% complete), also virtually absent from the other LCAs, and putatively gained from donors from Clostridales and from an unknown member of Bacteria.

## Discussion

### Definition of *Blautia* Genomic Species Groups Using Genomic Parameters

The comparative analysis of a set of 224 *Blautia* genomes presented here is the first genomic and taxonomic survey focused on this genus. This study found a set of 17 previously undescribed species, along with confirming the definition of 12 previously established species. These intra-species classifications were made on the basis of genomic metrics such as ANI, TETRA, and AAI values, according to previous standards (see below). In some cases, one parameter may have one value inside the intra-species range, but another one may not (e.g., some pairwise comparisons had TETRA > 0.99 but ANI < 95). This behavior was previously observed in other studies. For example, in the study where ANI was proposed as an standard for genomic classification (Richter and Rosselló-Móra, 2009), the comparison of ANI with other techniques such as DNA-DNA hybridization (DDH) or TETRA showed that, for a few cases, these methodologies had discrepancies with ANI when they were applied to formulate inter-species relationships. In addition, a high-scale study validating the use of ANI on a large scale (∼ 8 × 10^9^ comparisons) showed the presence of a “discontinuity zone” in the distribution of ANI values, where a very low fraction (∼ 0.2%) of ANI values were found between 95 and 83% (Jain et al., 2018), suggesting that this algorithm is robust to establish taxonomic relationships between genomes. This behavior was also observed in the distribution density plot for ANI values obtained in the *Blautia* dataset, with only a small fraction (<1%) in the “discontinuity zone” (Supplementary Figure 1F). Since ANI is one of the more commonly used metrics for defining new species from genomic data (Chun et al., 2018), it was selected as the main value to consider in the formulation of *Blautia* genomic species groups.

In several studies, one important form to define intra- and inter-species boundaries is the comparison of ANI or AAI versus % identity of the 16S rRNA gene (Kim et al., 2014). This, however, cannot be always possible, since several genomes, even with high quality, according to conventional metrics [based on CheckM (Parks et al., 2015), or BUSCO (Seppey et al., 2019)], do not have enough long 16S rRNA genes (>1,400 bases) to make appropriate comparisons. Since this limitation, results for this method were not reported in this study. Instead, we proposed to use a combination of similarity-based (AAI, ANI) and composition-based (TETRA) comparisons.

The analysis obtained 33 potential genomic species (i.e., species defined by genomic parameters), but some type strain genomes were found to belong to the same genome species group as other type strains. The two cases presenting this behavior are the pair *B. coccoides*-*B. producta* and the pair *B. hominis* - “*B. marasmi*”. In the first case, both species were reclassified in the same work (Liu et al., 2008), showing quite short distances between the representative 16S rRNA genes in the phylogeny. Those species had long different histories (*B. coccoides* was isolated circa. 1976 and *B. producta*, circa. 1941), and in combination with the absence of genomic data, those strains were not properly classified as members of the same species. Considering the information presented in this work, we proposed the name of this genomic group as *B. producta*, since the basonym of *B. producta* is older than those from *B. coccoides*. In the case of the *B. hominis*/*marasmi* pair, this genomic species remained as *B. hominis*,since “*B. marasmi*” is not an accepted name.

Several sequenced strains from this dataset contained species names that were not coincidental with their final assignment. For example, strains *Blautia coccoides* YL58 and *Blautia producta* SCSK (unknown group Y, see Supplementary Table 4 and Figure 1) were assigned to a potential new genomic species, different from the *B. producta/coccoides* group. Whereas their existing species name assignments were originally made on the basis of the 16S rRNA sequence identity, the combination of current genomic information generated by this study suggests that YL58 and SCSK strains can be classified as a potential new species. Since those strains were mistaken as *B. producta*/*B. coccoides*, they could be designated as “*Blautia pseudococcoides.*” In the case of other unknown groups, future projects covering already cultured or newly isolated strains must be carried out to study those potential new species with more detail, in order to confirm other properties (e.g., phenotype) of those variants and expand the knowledge about the *Blautia* genus.

### About the Pangenome of *Blautia*

The *Blautia* dataset has a core-to-pangenome ratio ∼ 1.9–2% (PEPPAN and panX), which at first may appear to be small. Depending on the lifestyle of the studied species and the sample size used to reconstruct the pangenome, this ratio can vary widely. For example, for P. *aeruginosa*, two studies displayed ratios of 15 and 9% (samples of 182 and 1360 genomes respectively) (Mosquera-Rendón et al., 2016; Park et al., 2019), while for *Shigella* spp., S. *pneumoniae* and S. *enterica* subsp. enterica this ratio was shown to be 2, 1.6, and 5.7% respectively (Park et al., 2019). Moreover, this dataset presents a proportionally big cloud genome, 87% of the pangenome, highlighting the extent of diversification that members of this genus have undergone. Related to this, we have shown the great amount of gene gain instances that appear to have occurred throughout evolution, therefore increasing the size of the current pangenome.

Considering the popularity of Roary (more than 1500 citations to Jan. 2021, Google Scholar), we investigated further to see if we could obtain similar results to the other tools. Roary, as a first step, uses a fast pre-clustering approach based on k-mers (CD-HIT) (Fu et al., 2012), followed by blastp (Camacho et al., 2009), and at this stage, it always found a relatively high amount of clusters, approximately 80 thousand. The cause of this particularly high number of ortholog clusters and thus the high number of distinct genes defining the pangenome, may relate to what has been shown recently by the authors of PEPPAN. They found that Roary made the highest number of false splits among the compared pipelines, where false splits were defined as the cases in which a single ortholog cluster in a curated pangenome was split into multiple ortholog clusters (Zhou et al., 2020). Related to the pre-clustering step performed by Roary, we note that we tried FindMyFriends too^3^ (results not shown), which also uses CD-HIT as a first step, and It failed to find any core genes.

It may be worthy of mention that the authors of Roary point out that their program is not intended for “comparing extremely diverse sets of genomes”^4^, although we are aware that It is not clear how to define which corresponds to a “diverse set of genomes” and we do not have any frame of reference to say that our dataset is particularly diverse.

### Role for Massive Gene Gain Events in the *Blautia* Evolution

This gene gain/loss analysis of a selected set of representative genomes of *Blautia* species suggested that gene loss events were minor forces during the evolution of the *Blautia* genus. Conversely, gene gain events seem to be frequent processes during the evolution of the *Blautia* genus, remarkably associated with metabolic functions. The results of the HGTector analysis suggest a set of remarkable donors for the predicted HGT events. However, when HGTector and Count data were compared, only a partial set of gained genes (between 30 and 40%) among different lineages could have a predicted donor. This discrepancy may be influenced by limitations in either the reference database or the algorithm used by HGTector, or by limitations of the model generated by Count, an aspect previously discussed in another study (Chen et al., 2021).

HGTector could find that, among selected genomes, most HGT predictions were associated with donors from the Firmicutes phylum, or other derived taxonomic groups, such as the *Ruminococcus* genus, the Clostridaceae family or the Clostridiales order (Figure 5). These findings have correspondence with three previously reported aspects: primarily, previous studies showed that HGT events tend to be shared among closely related species (Bolotin and Hershberg, 2017); secondly, it was also reported that HGT is very frequent in the GI tract for example, as seen in the human microbiome (Liu et al., 2012); finally, Firmicutes is known to be one of the most diverse and abundant groups in the mammalian GI tract microbiome (Youngblut et al., 2019). Considering these three aspects, the high percentage of Firmicute-related donors in the predicted acquired genes among different *Blautia* lineages is something completely expectable.

The results from this study not only suggest the presence of genes acquired or missed in the terminal nodes (reflecting the most recent gene gain/loss events in *Blautia* genomes), but also predicted gain/loss events from the predicted ancestors (Figure 4). Some functional differences were found in the gene functional profile of those gained in the LCA of each lineage, in comparison with those more recently gained. In the LCA of several lineages, most gain/loss events were associated with metabolic functions (Supplementary Figure 6). Instead, most gained genes in terminal nodes were unassigned to COG categories. This considerable proportion of gained and missed genes in the LCAs from the internal lineages associated with Metabolism-related functions may reflect that different *Blautia* lineages (especially, II) evolved primarily focusing in the adjustment of their respective metabolic abilities, involving a dynamic exchange of metabolic functions. These functional changes in the gene gain/loss patterns in the representative set was also observed in the comparison between predicted metabolic properties from different representative genome species clusters from the *Blautia* dataset (Figure 3).

Since experimental studies suggested that acquisition of auxotrophic features can be associated to natural selection and not merely to gene drift (D’Souza and Kost, 2016), it is possible that the dynamic metabolic gene gain/loss process observed in *Blautia* could reflect evolutionary pressures during the formation of the ancestors. Moreover, a comparative study with ∼1,000 genomes (Goyal, 2019) showed that bacterial genomes tend to lose previous pathways if they acquired new, alternate pathways by HGT, creating a dependency to coexisting bacteria whose pathways can compensate the changes in the evolved organism. In the case of *Blautia* evolution, metabolic function gain and loss may be provoked by the evolutionary context of each genomic species or lineage. For example, Lineage II (containing the species *B. hominis*, *B. coccoides/producta*, “*B. celeris*,” and unknown group Y) contained an acquired gene family encoding a butyrate kinase that is virtually exclusive from this group (data not shown). This enzyme is the final step in a butyrate biosynthesis pathway present in the Lachnospiraceae (Vital et al., 2014). This correlates with previous studies describing *B. coccoides* and *B. producta* as strong butyrate producers (Barcenilla et al., 2000). Therefore, the massive gene gain events in this lineage could reflect previously reported metabolic specializations.

### Final Conclusions and Future Projections

The analysis of a set of *Blautia* genomes from Refseq showed a previously unconsidered diversity of genomic species, finding 17 new potential species from the comparison of their genomes. This study also found that some previously accepted species require a reclassification on the basis of the genomic data. According to the information provided in this study, the type strain of *B. coccoides* is part of the *B. producta* species. Moreover, this study also generates evidence for the proposal of a new species, *B. pseudococcoides*, formerly classified as a *B. coccoides* strain (YL58). As far as we know, this is the first study covering aspects of the evolution of *Blautia* genetic content, opening the possibility for further studies focused on other aspects of *Blautia* metabolism.

There are some limitations to consider in future studies: since the vast majority of the genomes of the *Blautia* dataset were obtained from sequencing projects focused on human samples, our study could not specialize in tracking associations between gene families and different hosts. Future efforts will be needed to create an evolutionary model covering the relationships between *Blautia* species and different mammalian hosts. This study also needs to be extended by the use of metagenome- assembled genomes (MAGs), in order to expand to even a deeper level the analysis of the pangenome of this relevant genus of the mammalian GI tract microbiome.

### Reclassification of *Blautia coccoides* as *Blautia producta* Subspecies *coccoides* and Emended Description of *Blautia producta*

On the basis of genomic parameters, such as ANI, TETRA and AAI, as well as by phylogenomic profiling, the type strain of this former species (ATCC 29236 = DSM 935 = JCM 1395 = NCTC 11035) is reclassified as a subspecies of *Blautia producta.* The type strain of *B. producta* (ATCC 27340^T^ = CCUG 9990^T^ = CCUG 10976^T^ = DSM 2950^T^ = JCM 1471^T^) is proposed to be described as *B. producta* subspecies *producta*.

### Description of “*Blautia pseudococcoides*” sp. nov.

*“Blautia pseudococcoides” (pseu.do.coc.coi’des. Gr. adj. pseudo -us- -um false; Gr. n. coccus a berry; Gr. n. eidos shape; N.L. adj. coccoides berry shaped*, falsely recognized as *B. coccoides*, a close relative). This strain was recognized as a different species from other members of the *Blautia* genus on the basis of genomic parameters, such as ANI, TETRA and AAI, as well as by phylogenomic profiling. The proposed type strain is YL58^T^ (= DSM 26115^T^), isolated from wildtype mice (Lagkouvardos et al., 2016). Another variant of this proposed species is strain SCSK, isolated from ampicillin-resistant microbiota of mouse subjects (Caballero et al., 2017).

## Data Availability Statement

The original contributions presented in the study are included in the article/Supplementary Material, further inquiries can be directed to the corresponding author/s.

## Author Contributions

JPC conceived the study. All authors analyzed the data, wrote the manuscript, and approved the content.

## Conflict of Interest

The authors declare that the research was conducted in the absence of any commercial or financial relationships that could be construed as a potential conflict of interest.

## Footnotes

1 ftp://ftp.ncbi.nlm.nih.gov/genomes/

2 https://github.com/dparks1134/CompareM

3 https://rdrr.io/bioc/FindMyFriends/

4 https://sanger-pathogens.github.io/Roary/

## Supplementary Material

Supplementary Figure 1 Main statistics of the pairwise *genomic* comparisons in the *Blautia* dataset. **(A–C)** Scatterplots representing pairwise comparisons for different combinations of genomic parameters for taxonomic classification using genomic data for the *Blautia* dataset for TETRA vs ANI **(A)**, TETRA vs AAI **(B)** and AAI vs ANI **(C)**. Point colors reflect if each value reflects one, both or none of the values reached the threshold for intraspecies boundaries: violet for comparison whose two parameters meet the intraspecies criterion; red or blue when only the X-axis or Y-axis values meet the intraspecies criterion, respectively. **(D–F)**. Density plots representing pairwise comparisons for genomic parameters for taxonomic classification using genomic data for the *Blautia* dataset for TETRA **(D)**, AAI **(E)**, and ANI **(F)**. Category colors: green for intra-species comparisons; red for extra-species comparisons.

Supplementary Figure 2 Maximum-Likelihood tree estimated from an alignment of 190 conserved single-copy orthogroups present in the 224 genomes of *Blautia* plus the outgroup. Asterisks represent internal branches with bootstrap support (UF-bootstrap) and approximate likelihood-ratio test values (SH-aLRT) greater than 90. For internal branches with lower support, their numerical values are displayed (SH-aLRT/UF-bootstrap). Support values for terminal or near terminal branches are not shown for esthetics reasons. Terminal node colors are associated with the genomic species clusters defined from the ANI, TETRA and AAI data (see Supplementary Table 3 and the text). Clades in colored boxes represent four selected genomic species clusters with the highest number of genomes, which were analyzed in Figure 3.

Supplementary Figure 3 Approximately-maximum-likelihood phylogenetic tree built by FastTree and refined by RaxML, as used in panX. This tree was built from the variable positions of a multiple sequence alignment of all single-copy core genes defined by panX using as input the 224 genomes of the *Blautia* dataset plus the outgroup.

Supplementary Figure 4 Comparison of the trees depicted in Supplementary Figures 1, 2. The comparison, made by the function *plot.cophylo* from the R library *phytools* (Revell, 2012), was built between the unrooted trees as they were originally produced by IQ-Tree and panX. Tips were rotated in order to build a more readable comparison, minimizing the number of crossing lines.

Supplementary Figure 5 COG functional annotations for the shell and core genomes of the *Blautia* dataset. The legend is common for both plots and is divided for space reasons. For the shell (left) and core genomes (right), 91 and 99.4% of the genes were designated under at least one COG category respectively (including category S, “Function unknown”).

Supplementary Figure 6 Heatmap of the completeness of KEGG modules in the core and shell genomes. Heatmap produced by MicrobeAnnotator (Ruiz-Perez et al., 2021), for annotations from the Kofam (Aramaki et al., 2020) and Uniprot (UniProt Consortium, 2019) databases. Only modules complete at least 50% in at least one genome are considered.

Supplementary Figure 7 Pie Chart representations for the COG meta-categories found in the gained and missed gene families at different LCAs from different lineages of the *Blautia* evolution, observed in the set of representative genomes (see Figure 4). Gene families were annotated using COG. Data for the LCA from lineages I to IV, as well as the total gene set acquired and missed in the terminal nodes, were represented.

Supplementary Table 1 Currently established/proposed *Blautia* species in September 2020.

Supplementary Table 2 List of the *Blautia* RefSeq genome assemblies utilized in this study.

Supplementary Table 3 Genomic pairwise calculations for ANI, TETRA, and AAI obtained from the *Blautia* dataset and the outgroup (GCF_014287855.1).

Supplementary Table 4 List of the genomic species clusters defined from the ANI + TETRA + AAI data explained in the text, and their respective members.
