# Supplementary figures and images for: Insights on the Evolutionary Genomics of the Blautia Genus: Potential New Species and Genetic Content Among Lineages
Source: Front Microbiol. 2021 Apr 26;12:660920. doi: 10.3389/fmicb.2021.660920 (PMC8107234; doi:10.3389/fmicb.2021.660920)

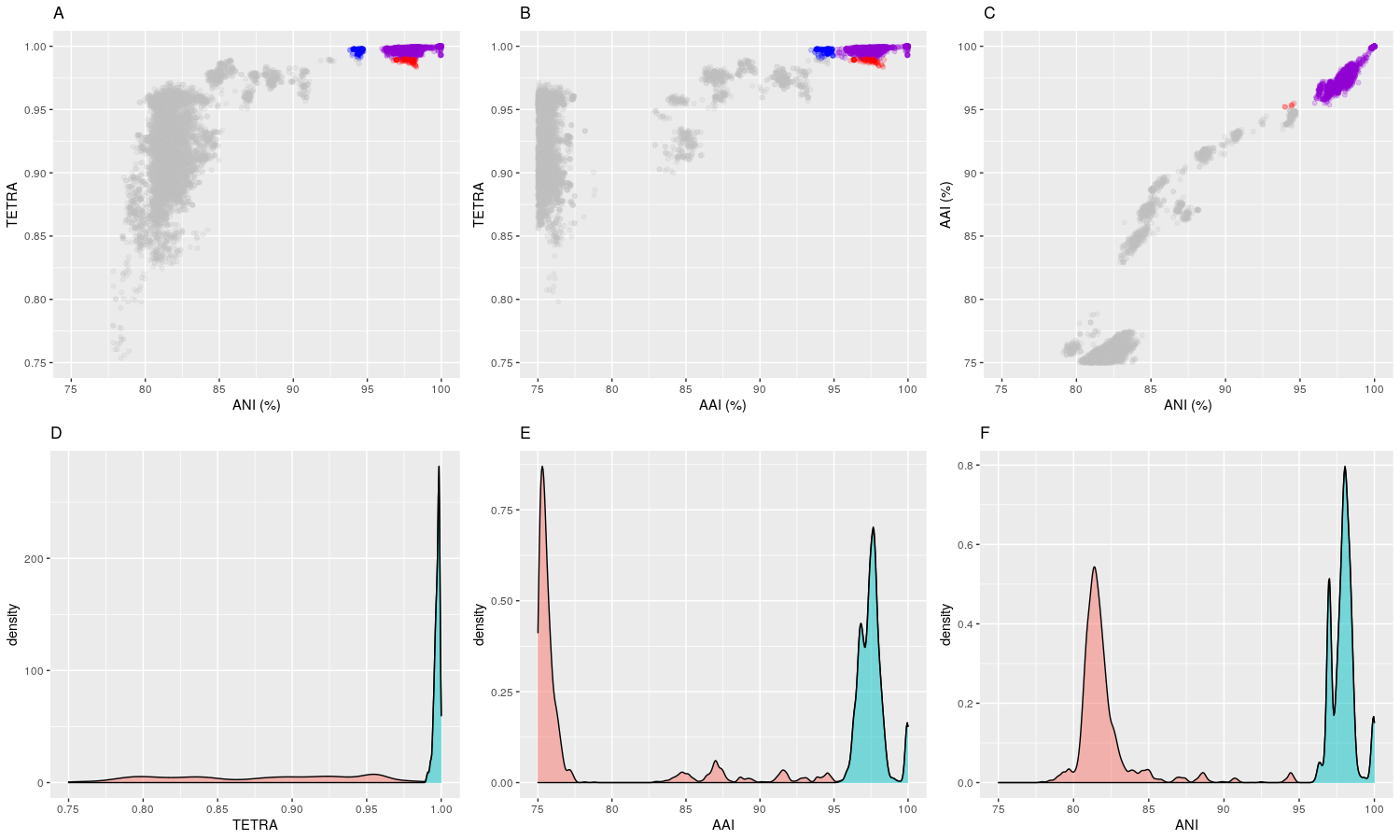

Supplement: Supplementary Figure 1 — Main statistics of the pairwise genomic comparisons in the Blautia dataset. (A–C) Scatterplots representing pairwise comparisons for different combinations of genomic parameters for taxonomic classification using genomic data for the Blautia dataset for TETRA vs ANI (A), TETRA vs AAI (B) and AAI vs ANI (C). Point colors reflect if each value reflects one, both or none of the values reached the threshold for intraspecies boundaries: violet for comparison whose two parameters meet the intraspecies criterion; red or blue when only the X-axis or Y-axis values meet the intraspecies criterion, respectively. (D–F). Density plots representing pairwise comparisons for genomic parameters for taxonomic classification using genomic data for the Blautia dataset for TETRA (D), AAI (E), and ANI (F). Category colors: green for intra-species comparisons; red for extra-species comparisons. [file Image_1.PNG]

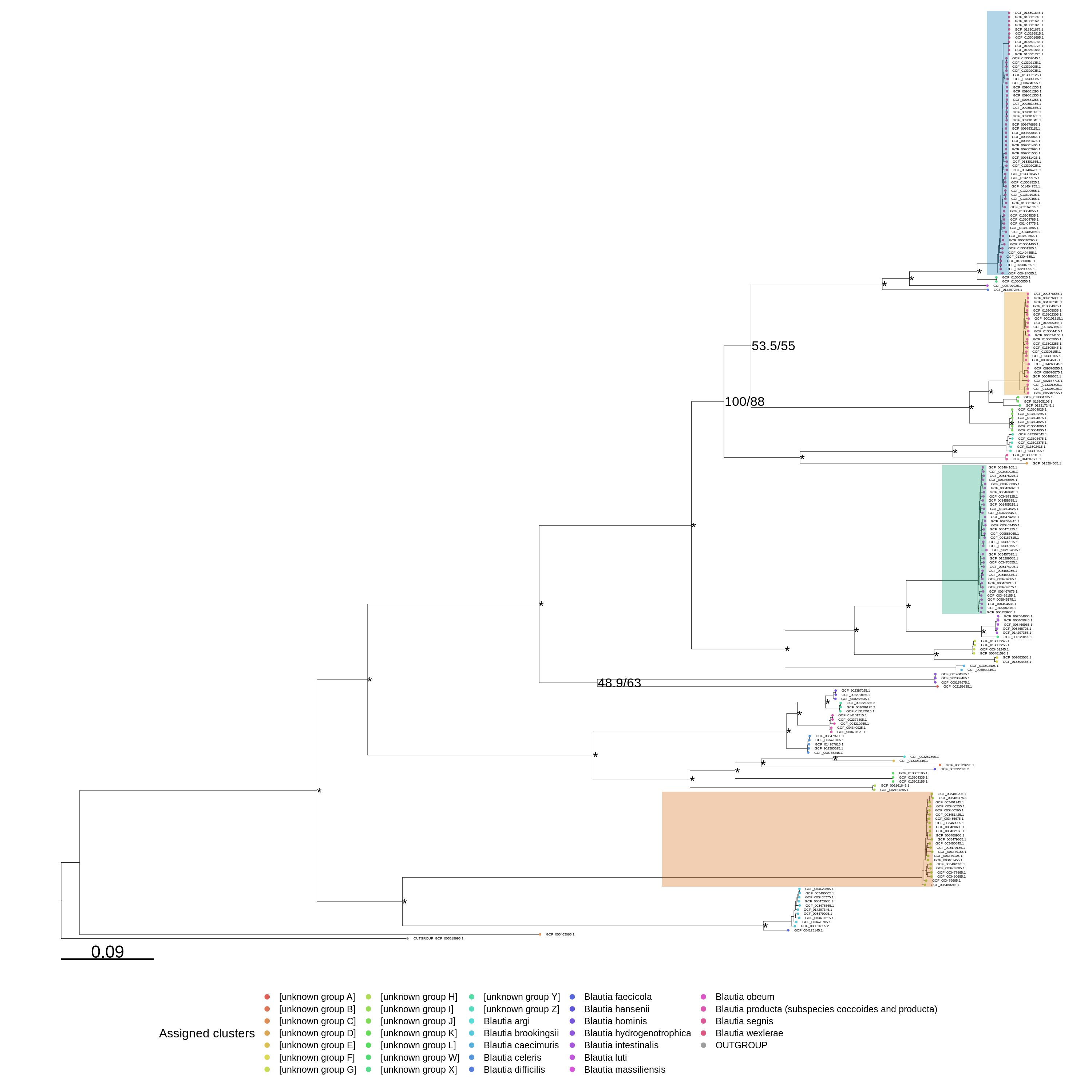

Supplement: Supplementary Figure 2 — Maximum-Likelihood tree estimated from an alignment of 190 conserved single-copy orthogroups present in the 224 genomes of Blautia plus the outgroup. Asterisks represent internal branches with bootstrap support (UF-bootstrap) and approximate likelihood-ratio test values (SH-aLRT) greater than 90. For internal branches with lower support, their numerical values are displayed (SH-aLRT/UF-bootstrap). Support values for terminal or near terminal branches are not shown for esthetics reasons. Terminal node colors are associated with the genomic species clusters defined from the ANI, TETRA and AAI data (see Supplementary Table 3 and the text). Clades in colored boxes represent four selected genomic species clusters with the highest number of genomes, which were analyzed in Figure 3. [file Image_2.PNG]

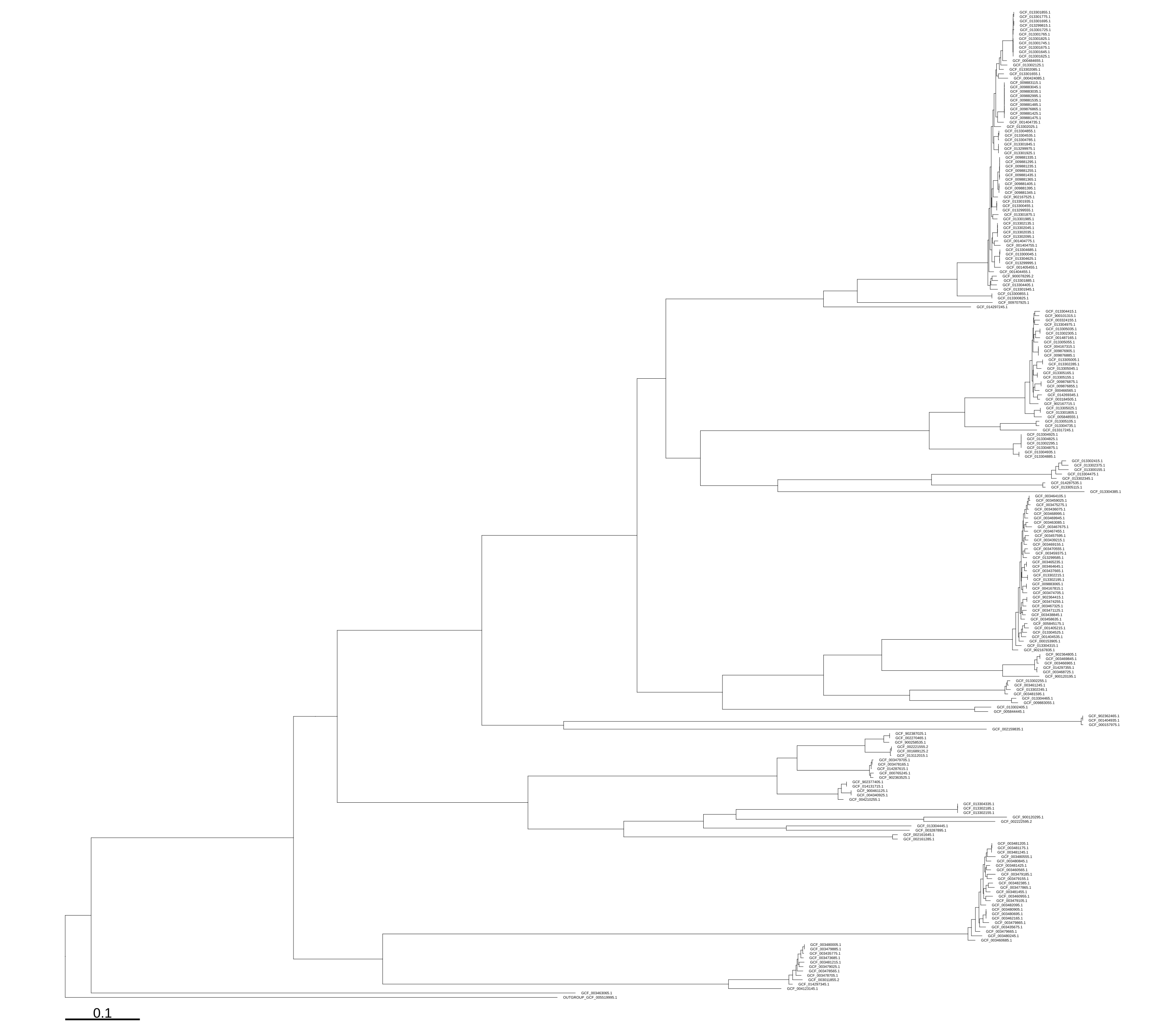

Supplement: Supplementary Figure 3 — Approximately-maximum-likelihood phylogenetic tree built by FastTree and refined by RaxML, as used in panX. This tree was built from the variable positions of a multiple sequence alignment of all single-copy core genes defined by panX using as input the 224 genomes of the Blautia dataset plus the outgroup. [file Image_3.PNG]

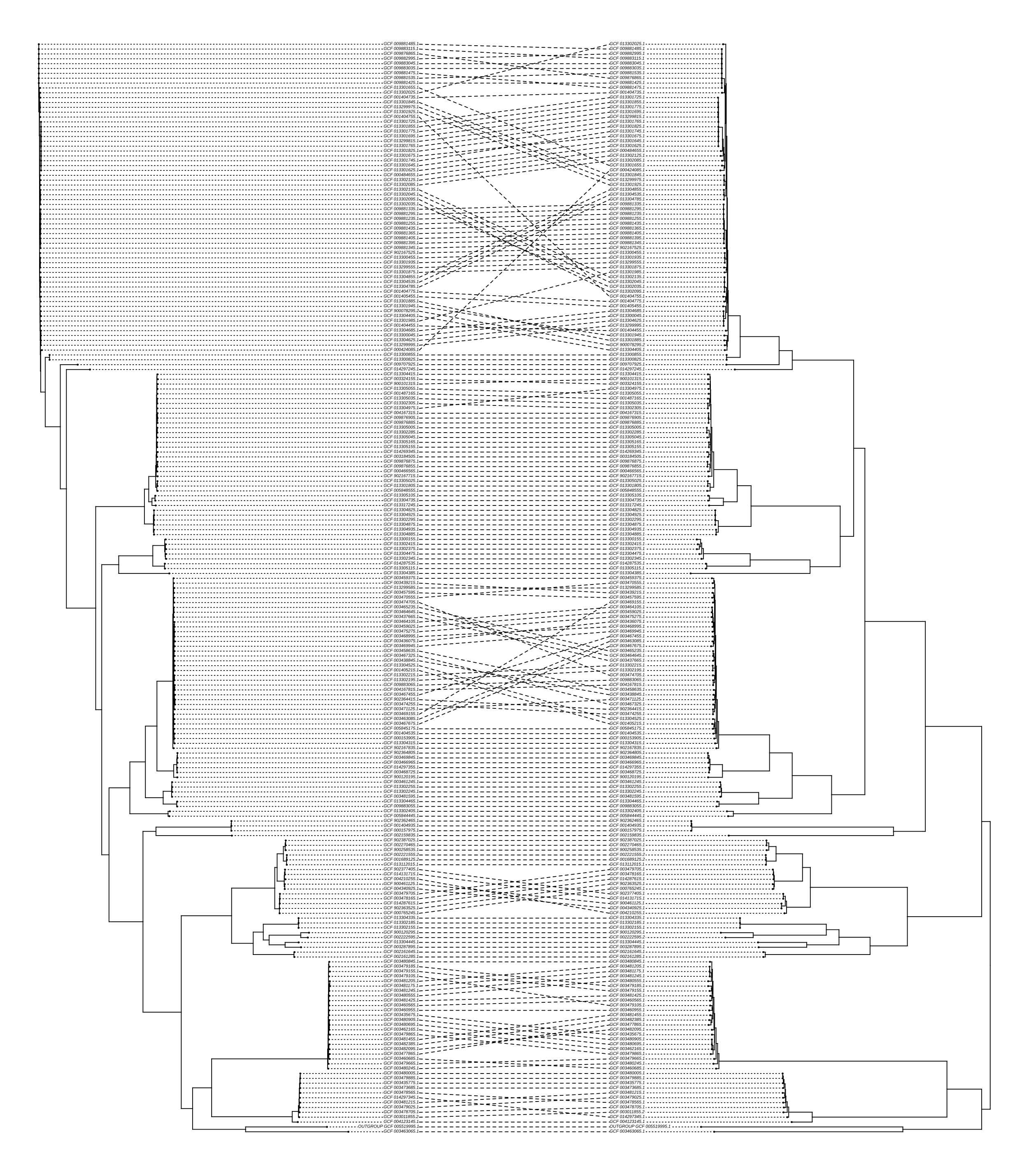

Supplement: Supplementary Figure 4 — Comparison of the trees depicted in Supplementary Figures 1, 2. The comparison, made by the function plot.cophylo from the R library phytools (Revell, 2012), was built between the unrooted trees as they were originally produced by IQ-Tree and panX. Tips were rotated in order to build a more readable comparison, minimizing the number of crossing lines. [file Image_4.PNG]

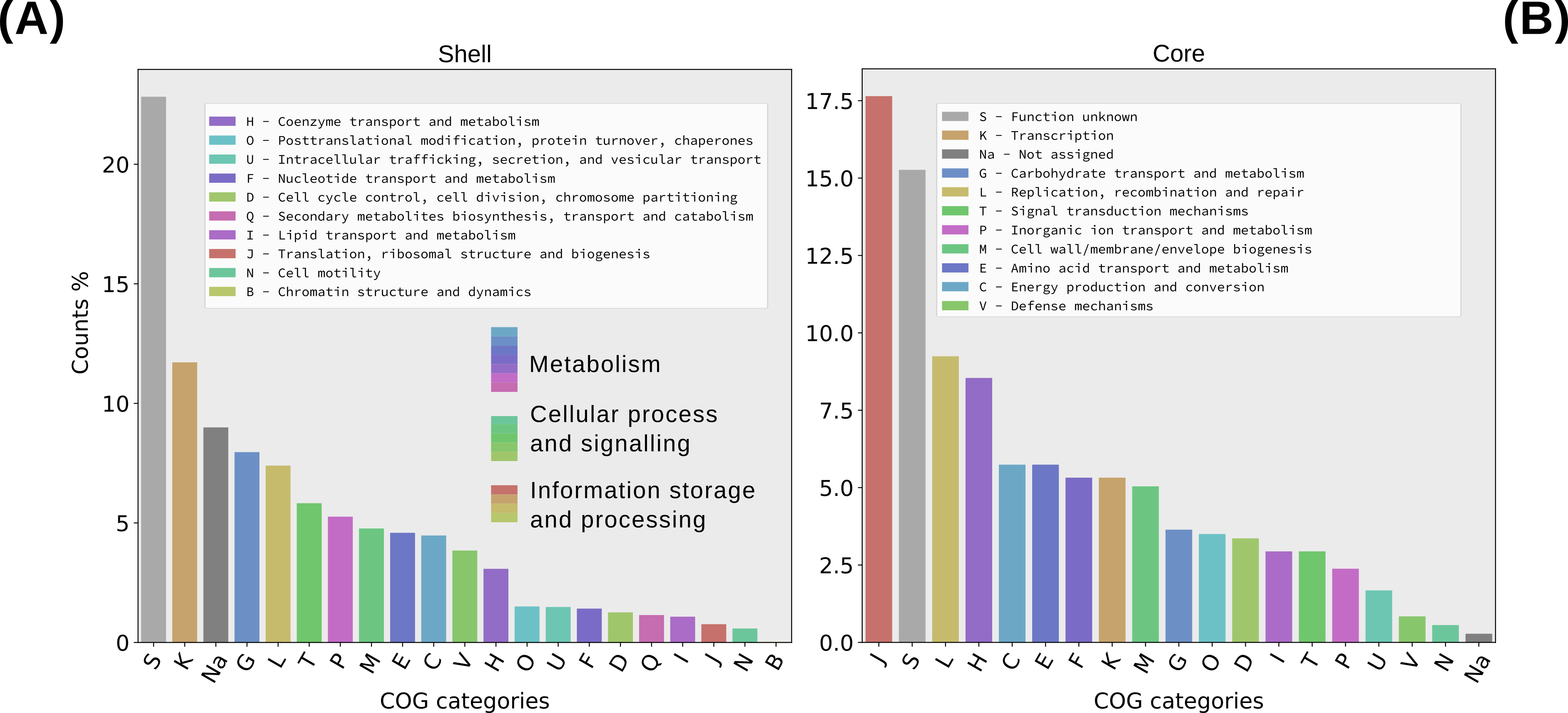

Supplement: Supplementary Figure 5 — COG functional annotations for the shell and core genomes of the Blautia dataset. The legend is common for both plots and is divided for space reasons. For the shell (left) and core genomes (right), 91 and 99.4% of the genes were designated under at least one COG category respectively (including category S, “Function unknown”). [file Image_5.PNG]

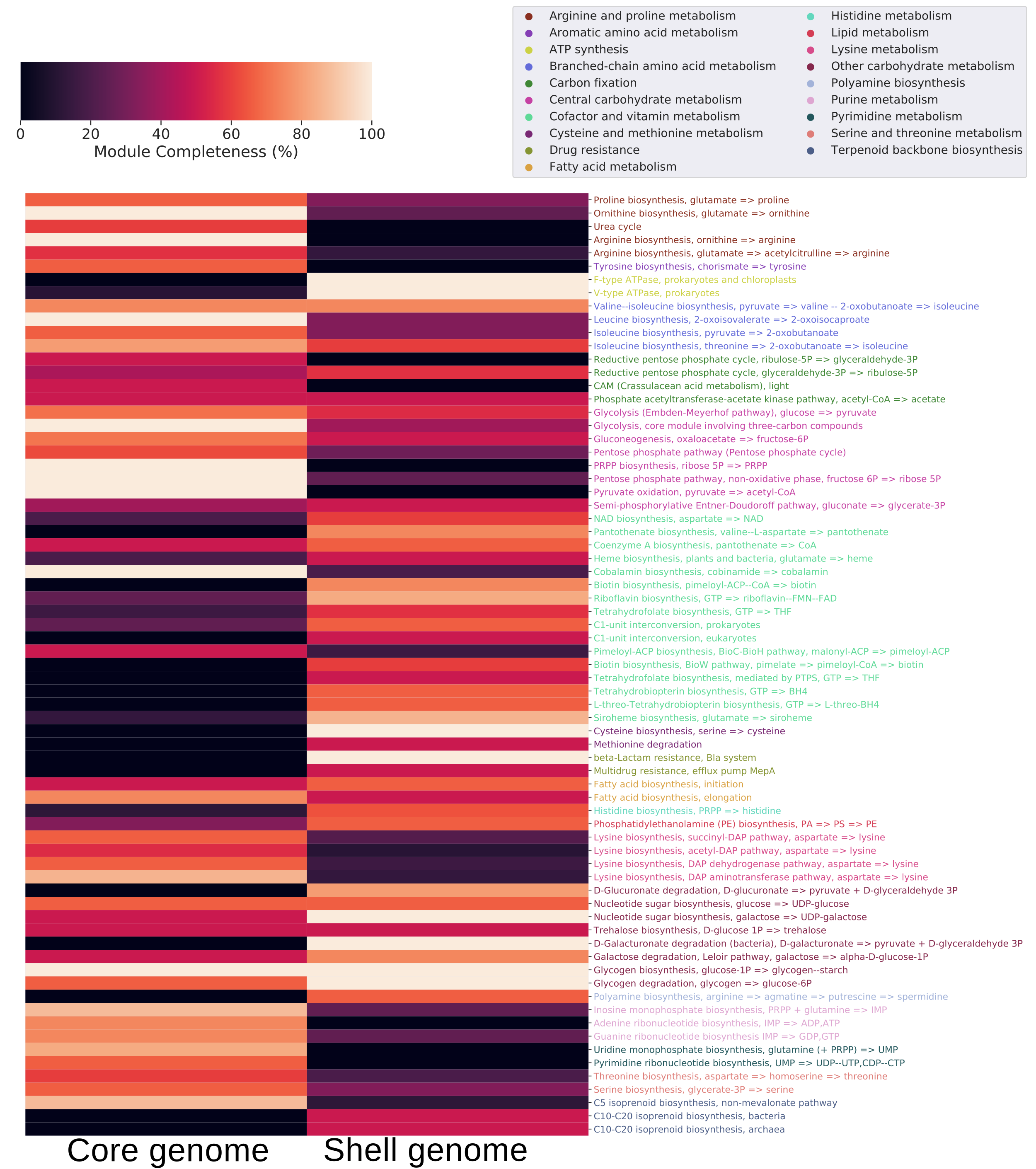

Supplement: Supplementary Figure 6 — Heatmap of the completeness of KEGG modules in the core and shell genomes. Heatmap produced by MicrobeAnnotator (Ruiz-Perez et al., 2021), for annotations from the Kofam (Aramaki et al., 2020) and Uniprot (UniProt Consortium, 2019) databases. Only modules complete at least 50% in at least one genome are considered. [file Image_6.PNG]

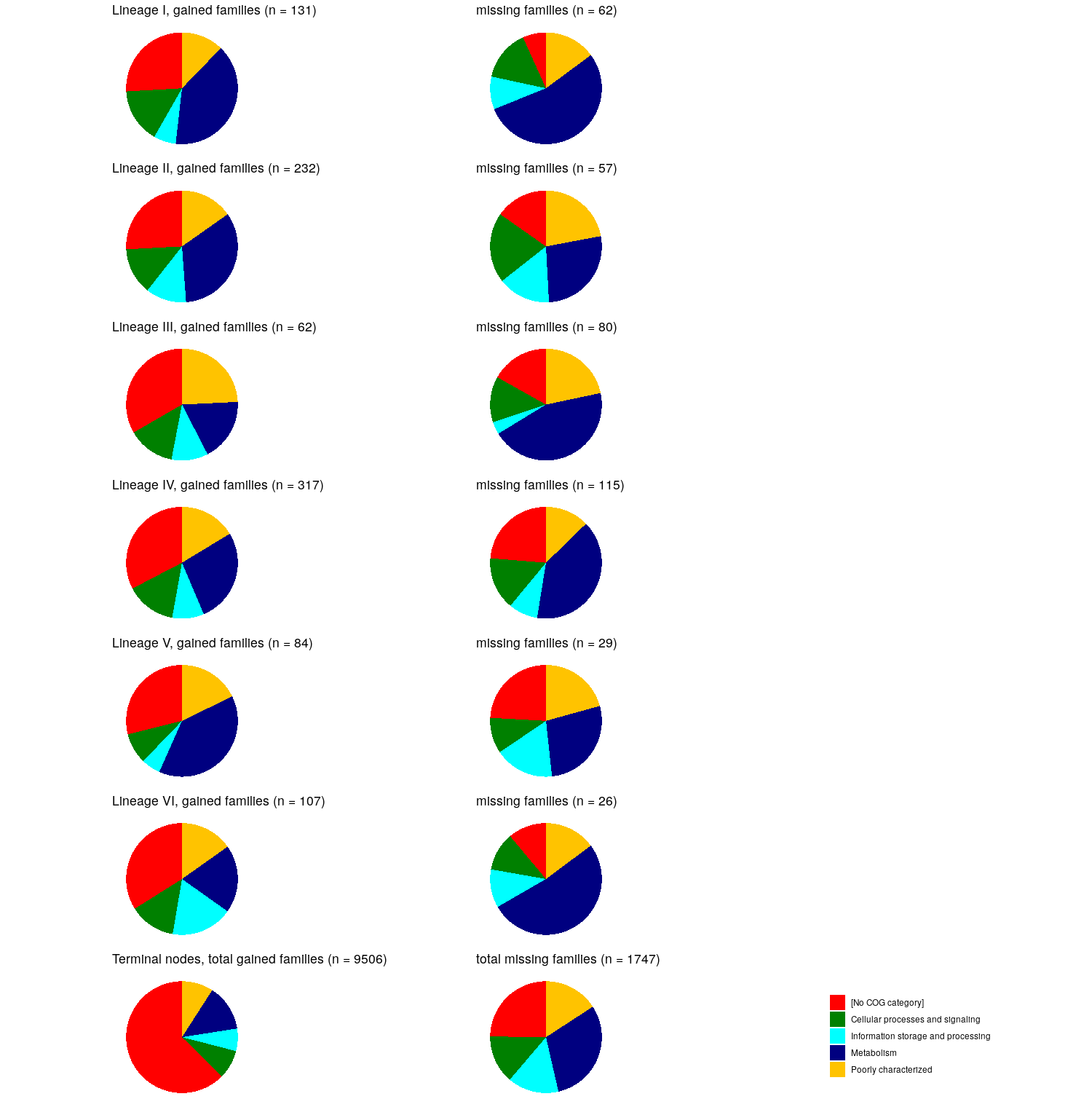

Supplement: Supplementary Figure 7 — Pie Chart representations for the COG meta-categories found in the gained and missed gene families at different LCAs from different lineages of the Blautia evolution, observed in the set of representative genomes (see Figure 4). Gene families were annotated using COG. Data for the LCA from lineages I to IV, as well as the total gene set acquired and missed in the terminal nodes, were represented. [file Image_7.PNG]
